# Alpha-tocopherol fertigation confers growth physio-biochemical and qualitative yield enhancement in field grown water deficit wheat (*Triticum aestivum* L.)

**DOI:** 10.1038/s41598-019-49481-7

**Published:** 2019-09-09

**Authors:** Qasim Ali, Shafaqat Ali, Naeem Iqbal, Muhammad Tariq Javed, Muhammad Rizwan, Roubina Khaliq, Sumreena Shahid, Rashida Perveen, Saud A. Alamri, Mohammed Nasser Alyemeni, Leonard Wijaya, Parvaiz Ahmad

**Affiliations:** 1 Department of Botany, Government College University, Faisalabad, 38000 Pakistan; 2 Department of Environment Sciences and Engineering, Government College University, Faisalabad, 38000 Pakistan; 3 Department of Physics, University of Agriculture, Faisalabad, 38040 Pakistan; 4 Department of Botany and Microbiology, College of Science, King Saud University, Riyadh, Saudi Arabia; 5 Department of Botany, S.P. College, Maulana Azad Road, Srinagar, Jammu and Kashmir 190001 India

**Keywords:** Plant signalling, Drought

## Abstract

Water stress is a major problem to fulfill the world food demand and to solve the problem of malnutrition. Different strategies are being used to solve these problems including the fertigation of plants with different biochemical at different growth stages. The present study was conducted for the induction of drought tolerance in field grown wheat for better yield and nutritional quality through foliar spray of α-tocopherol (α-Toc) at start of reproductive stage. Water stress was maintained based on number of irrigation. Three levels of α-Toc 0.001, 0.01 and 0.1 mM were applied as foliar spray. Water stress significantly reduced the biomass production that associated with the decreased photosynthetic pigments, water relation, photosynthetic efficiency, but increased the lipid peroxidation, leaf relative membrane permeability, activities of antioxidant enzymes and the contents of phenolic, flavonoids, α-toc and ASA. Water stress also negatively effected the different yield attributes and seed nutrient quality. Foliar fertigation of wheat plants with α-Toc significantly improved the water stress tolerance of wheat plants in term of improvement in growth and seed yield associated with improved water relations, photosynthetic efficiency, contents of photosynthetic pigments and improvement in antioxidative defence mechanism (enzymatic and non-enzymatic antioxidants). Fertigation of water stressed wheat plants with α-Toc also improved the seed nutritional quality in terms of the contents of seed phenolics, flavonoids, activities of antioxidant enzymes and the content of α-, β- and γ-tocopherols. In conclusion, it was found that fertigation of water stressed wheat plants not only improved the water stress tolerance but also improved the seed yield and nutritional quality that will not only be helpful for the improvement in wheat yield that also be a step to solve the problem of malnutrition through the bio-fertification of α-Toc.

## Introduction

Present world scenario about shortage of fresh water in agriculture and further reports about shortage of water for agriculture in near future has created a problem for farmers to obtain better crop production along with better nutritional quality. Along with shortage of fresh water, a rapid increase in world population has further created a threat and challenging condition to fulfill world food demand for the agricultural researchers. Due to global warming, increase in mean world temperature has significantly changed the rainfall pattern and creating a problem of aridity with a drop in crop production. Drought stress adversely effects plant life by creating perturbations primarily in plant water relation, photosynthetic activity, creating oxidative stress as a result reduced productivity.

Different ways and strategies are being used and further being explored to counteract this problem for better yield^1–3^. These include the production of stress tolerant genotypes or the induction of stress tolerance through exogenous use of different organic compounds such as different metabolites including varying vitamin, growth regulators, osmoprotectants, chemical messenger as well as antioxidant. The sensitivity of plants to water shortage is growth stage and species/cultivars specific. Among different growth stages the reproductive stage is most sensitive one that severely effects the final production. The better ameliorating affects by the exogenous use of different chemicals depends on the concentration of the chemical and the growth timing of the plant. It is well known that these chemicals are actively taken up by the plants after their application, translocate to different plant parts where they take part in different cellular metabolic activities by boosting up the photosynthetic mechanism, through maintaining plant water relation by cellular osmotic adjustment and cellular antioxidative defense mechanism improvement and as a result better yield. It has been found that exogenous application of these compounds along with growth and yield improvements also found effective in improving the seed nutritional quality^4–6^.

Along with other chemicals tocopherols are also include in the list of such chemicals that found effective for induction of stress tolerance in plants through its internal accumulation or through its exogenous application^4–6^. It is a family of lipid soluble compounds, which have great role in different cellular activities. These include the tocopherol and tocotrienols. Among them α-Toc is consider the most important one. The important tocochromanol that are present in leaves are tocopherol^7^. Tocopherol (vitamin E) is a lipid-soluble antioxidant synthesized only by all plants and is an essential part of human nutrition and health. Its levels are tissue specific and mainly fluctuate under stressful conditions^8^, where it actively takes part in different metabolic activities^9^. Mainly they are present in the chloroplast of leaf as a defensive compound. It plays a major role in ROS scavenging, membrane stabilization while interacting with the polyunsaturated acyl groups of lipids^10^ and protects polyunsaturated fatty acids from lipid peroxidation and regulates varying signal transduction pathways^11^. Tocopherols perform like a terminator in chain reaction for polyunsaturated fatty acids removal^12^ by scavenging and quenching of oxygen^13^. Where they show significant defensive responses in different stresses especially in abiotic ones (salt, drought, & light etc.) and provide protection against oxidation damages to defend plant chloroplast membrane^14^ and to maintain the integrity of chloroplast.

Alpha tocopherol has been found to play various metabolic roles in plants through endogenous synthesis or through the exogenous application^4–6^. It was found that leaf transpiration and respiration rate affected significantly with the level of cellular tocopherol that effectively improved the tolerance to various stresses^14^. In sunflower enhanced photosynthesis was found after tocopherol application that was associated with decreased ABA content^15^. Similarly in sunflower and faba bean α-Toc improved the leaf chlorophyll content^16^.

Enhanced accumulation of carbohydrates was found in maize plants under water stress by tocopherol application that was associated with its involvement in increments in phytoharmones^15^. However, the stress tolerance improving effects of α-Toc are plant species specific. For example^17^ reported an increase in growth of sunflower plants that was associated with the improvement in photosynthetic pigments and carbohydrate accumulation but in rice plants no improvement in growth and yield was found after α-Toc applications^18^. In *Pelargonium graveolens* L. plants α-Toc application improved the yield and essential oil production^19^ with an improved lipid peroxidation.

Though tocopherols are found effective for the induction of stress tolerance in crop plants but its roles in yield and nutritional quality increments are still lacking especially at specific growth stages. Secondly, the studies presented here are mostly showing the effects of α- Toc applications at vegetative stage and lacking at the reproductive stage. It was hypothesized that exogenous use of tocopherol at reproductive stage might improve the nutritional quality through its ameliorative effects on antioxidative defense mechanism.

The aim of the study to find out the role of foliar applied different levels of α-Toc in the induction of drought tolerance in view of its role in plant photosynthetic activity and water relations in relation with the increment in final grain yield and nutritional quality.

## Materials and Methods

The experiment was conducted in field during wheat growth season at wheat research institute, AARI, Faisalabad under shed having movable shelter for the protection of field from rain, to access the influence of foliar-applied α-Toc on water stressed wheat plants at heading stage. The seeds of wheat cultivars Punjab 11 were obtained from seed bank of wheat research section of AARI, Faisalabad, Pakistan. The total experimental area was comprised a main plot divided into two sub-plot, each nominating the specific water stress level (control and drought). The experiment was carried out in randomized complete block design (RCBD) with three replications of each treatment. Before sowing of seeds the soil was well prepared as per the conditions required for wheat. The soil was prepared by ploughing field after 15 days of irrigation when the soil was at the proper field capacity for sowing of seeds. Seeds were sown in rows. Each row was 20 ft long with 6 inches row to row distance. Seeds were hand sown by making holes with the help of a dobbler with a seed to seed distance of 3 inches. The first irrigation to both plots was applied after 15 days of seed germination. A total four irrigations were applied to the non-stressed plants but the water stressed plants were supplied with second irrigation at the late vegetative stage and no irrigation was applied after the second irrigation.

Four levels of α-Toc (0, 0.001, 0.01, 0.1 mM) were applied as foliar spray at the heading stage. The solution of α-Toc for foliar spray was prepared by dissolving it in a minimal amount of methanol and then made the final required volume using distilled water. Foliar application of α-Toc was done in evening before sunset for its maximum uptake of leaf. Before foliar application each prepared solution was supplied with Twin-20 (0.1% was added as surfactant) for the maximum absorption. Data for various studied attributes was collected after 15 days of α-Toc spray. The parameters such as shoot and leaf fresh mass, flag leaf area, water potential and leaf relative water content were measured at the experimental site while the dry masses of shoot and leaf were measured in lab after oven drying the materials. The other biochemical attributes were estimated in experimental botany lab, department of botany, Government College University, Faisalabad.

### Soil chemical composition of experimental areas

The soil of the experimental area was sandy clay having average 25% sand, 15% silt, and 60% clay content. The method described by^20^ was used to determine the soil texture using hygrometer. The organic matter in soil was 0.81%, with a saturation percentage of 32%. On dry weight basis, the soil was comprised NO_3_-N 7.1, NH_4_-N 3.50, available phosphorous 7, calcium 112 and potassium 200 (all values of nutrients in mg/kg of dry soil). The pH of the soil was 8.1 and the ECe was 2.1 dS/m. The method described by^21^ was employed for the estimation of pH, ECe, and the inorganic nutrients.

### Averaged meteorological conditions during the course of experiment

#### Estimation of growth and physiological attributes

Shoot and leaf fresh masses were measured using an electric balance and the dry masses were measured after oven drying the fresh material at 70 °C for three days. Flag leaf area was measured following the method of^22^ using following formula:

$${\rm{Flag}}\,{\rm{leaf}}\,{\rm{area}}={\rm{leaf}}\,{\rm{length}}\times {\rm{leaf}}\,{\rm{width}}\times 0.75$$

*Here 0.75 is the correction error (CE) for monocots.

#### Estimation of gas exchange characteristics

Infrared gas analyzer (C-340, CID, INC, USA) was used to record the different gas exchange characteristics. The time of readings was 9:00 a.m. to 12:00 p.m. during appropriate weather conditions and during this photosynthetic active radiation was ranging between 587 to 1569 µmolm^−2^s^−1^. The different studied attributes were include the flag leaf net photosynthesis (*A*), flag leaf transpiration rate (*E*), water use efficiency (WUE = *A*/*E*), stomatal conductance (*g*_*s*_), flag leaf internal carbon dioxide (*Ci*) and internal CO_2_/atmospheric CO_2_ (*Ci*/*Ca*).

#### Leaf water potential

From the top of the plant expanded leaves were excised with a blade. Cut the leaf from its end of midrib very little extruding out and was placed in the pressure chamber. Valve of compressed gas cylinder opened slowly to increase pressure. Drop of xylem sap from the cut end of the midrib was watched from magnifying glass. When the drop of xylem sap was appeared then valve of compressed gas immediately closed and reading was recorded from the gauge of pressure chamber. Reading obtained from pressure chamber was in bars, converted them into MPa by dividing the reading with ten.

#### Leaf relative water contents (LRWC)

A fully developed young leaf (3^rd^ most from top) from each replicate was used to determine the LRWC. Fresh weight of leaf was measured and marked with specific tag using permanent marker. Then the leaf was dipped in distilled water for 4 h and find out the leaf turgid weight after absorbing access water on leaf surface using a blotting paper. Then the dry weight of leaves was determined after drying in an electric oven at 70 °Ċ for 48 h. Then the following formula was used to determine the LRWC on % basis:

$${\rm{LRWC}}\,( \% )=\frac{{\rm{Leaf}}\,{\rm{fresh}}\,{\rm{weight}}-{\rm{Leaf}}\,{\rm{dry}}\,{\rm{weight}}}{{\rm{Leaf}}\,{\rm{turgid}}\,{\rm{weight}}-{\rm{Leaf}}\,{\rm{dry}}\,{\rm{weight}}}\times 100$$

#### Leaf chlorophyll and carotenoid contents

The contents of leaf photosynthetic pigments such as chlorophyll (Chl.) *a*, *b*, total Chl. and Chl. *a/b* were estimated following^23^. The contents of carotenoids were estimated following^24^. The extraction of photosynthetic pigments was done using 80% acetone. The absorbance of the extract was read at 663, 645 and 480 nm using spectrophotometer (Hitachi U-2001, Tokyo, Japan). The quantities were computed using the specific formulas:

$${\rm{Chl}}{\rm{.}}\,a=[12.7\,({\rm{OD}}\,663)-2.69\,({\rm{OD}}\,645)]\times {\rm{V}}/1000\times {\rm{W}}$$

$${\rm{Chl}}{\rm{.}}\,b=[22.9\,({\rm{OD}}\,645)-4.68\,({\rm{OD}}\,663)]\times {\rm{V}}/1000\times {\rm{W}}$$

$${\rm{Total}}\,{\rm{Chl}}=[20.2\,(\Delta {\rm{A}}\,645)-8.02(\Delta {\rm{A}}\,663)]\times {\rm{v}}/{\rm{w}}\times 1/1000$$

$${\rm{A}}\,{\rm{carotenoid}}\,(\mu {\rm{g}}/{\rm{g}}\,{\rm{FW}})=\Delta {\rm{A}}480+(0{\rm{.114}}\times \Delta {\rm{A}}663)-(0.638\times \Delta {\rm{A}}645)$$

$${\rm{Carotenoids}}={\rm{A}}\,\mathrm{car}/\mathrm{Em}\,100 \% \times 100$$

$${\rm{Em}}=({\rm{Emission}})={\rm{Em}}\,100 \% =2500$$

$$\Delta {\rm{A}}={\rm{absorbance}}\,{\rm{at}}\,{\rm{respective}}\,{\rm{wavelength}}$$

$${\rm{V}}={\rm{volume}}\,{\rm{of}}\,{\rm{the}}\,{\rm{extract}}\,({\rm{mL}})$$

$${\rm{W}}={\rm{weight}}\,{\rm{of}}\,{\rm{the}}\,{\rm{fresh}}\,{\rm{leaf}}\,{\rm{tissue}}\,({\rm{g}})$$

#### Leaf MDA

Leaf MDA content as the extent of lipid peroxidation was aescribed following the method of^25^ with slight modifications. Leaf samples of 1.0 g were homogenized in 3 mL of 0.1% (w/v) trichloroacetic acid (TCA) solution. The homogenate was centrifuged at 20000 × *g* for 15 min. Three mL of 0.5% thiobarbituric acid (TBA) prepared in 20% TCA were added to 0.5 mL of the supernatant. The mixture was heated at 95 °C in a shaking water bath for 50 min. The reaction was stopped by cooling the tubes in a water bath containing chilled water. Then the samples were centrifuged at 10,000 × *g* for 10 min, and the absorbance of the supernatant was read at 532 and 600 nm. The MDA content was calculated as the difference in absorbance at 600 and 532 nm using the following formula:

$${\rm{MDA}}\,{\rm{level}}\,({\rm{nmol}})=\Delta \,({\rm{A}}\,532\,{\rm{nm}}-{\rm{A}}\,600\,\mathrm{nm})/1.56\times 105$$

Absoption coefficient for calculating MDA is 156 mmol^−1^cm^−1^.

#### Leaf H_2_O_2_

H_2_O_2_ content was assayed by following the protocol reported by^26^. Plant samples were homogenized in TCA. 0.1 ml of enzyme was taken and 1 ml of KI was added it. The absorbance of the mixture was measured at 390 nm. The H_2_O_2_ content was estimated using a standard curve generated from a series of standard solutions with known concentrations of pure H_2_O_2_.

#### Antioxidant enzyme extraction in leaf and seed

For the extraction of antioxidant enzymes, frozen fresh leaf material (0.5 g) was homogenized in ice cold buffer solution (Na-phosphate buffer pH 7.0) containing 100 mM Tris (pH 7.0), 10 mM d-isoascorbic acid, 20 g L^−1^ PVP-10, 1.5 g insoluble PVP, 0.1 mM EDTA and 2 mL L^−1^ Triton X-100. Estimation of quantitative TSP was executed by method of^27^, using bovine serum albumin (BSA) as standard.

### Activities of SOD and POD in leaf

#### SOD activity

The activity of SOD was measured by monitoring the inhibition of photochemical reduction of nitro-blue tetrazolium (NBT) at 560 nm according to the method described by^28^. The activity of SOD was determined by adding 50 μl of the enzymatic extract to a solution containing (total reaction solution including enzyme extract 1 mL) 50 μM NBT (NBT dissolved in ethanol), 1.3 μ*M* riboflavin, 13 m*M* methionine, 75 n*M* EDTA, 50 m*M* phosphate buffer (pH 7.8), and 50 μl enzyme extract. The reaction solutions were kept in a chamber having internal side coated with aluminum under illumination of fluorescent lamps of 30 W. The reaction was started by turning the fluorescent lamps on, and stopped 5 min later by turning them off. The blue formazane produced by NBT photoreduction was measured as increase in absorbance at 560 nm. The reaction mixture lacking leaf extract was taken as control and kept in light. The absorbance of the irradiated solution was read at 560 nm using a UV-visible spectrophotometer (IRMECO U2020). One unit of SOD was defined as the amount of enzyme required to cause 50% inhibition of the rate of NBT reduction at 560 nm in comparison with tubes lacking the plant extract.

#### POD activity

The guaiacol oxidation method^29^ was used for the estimation of POD activity. The reaction mixture (3 ml) contained 0.1 ml enzyme extract, 50 mM phosphate buffer (pH 7.0), 20 mM guaiacol and 40 mM H_2_O_2_. Change in absorbance of the resulting mixture was read at 470 nm after every 20 s for 180 s.

#### Catalase activity

Catalase activity (CAT) was determined by using the method of^30^ with some modifications. Reaction solution (3 mL) of CAT was comprised of 50 m*M* phosphate buffer (pH 7.8), 5.9 mM hydrogen peroxide and 0.1 mL enzyme extract. Reaction was initiated by the addition of H_2_O_2_ to the reaction solution. For 3 minutes, after every 20 seconds, the CAT activity was noted by measuring the decrease in absorbance at 240 nm using spectrophotometer. One unit of POD activity was defined as an absorbance change of 0.01 U min^−1^.

#### Ascorbate peroxidase (APX) activity

The APX activity was determined following the method as aescribed by^31^. The reaction mixture (1600 µL) containing 50 mM potassium phosphate buffer (pH 7.0), 0.5 mM ascorbic acid, 0.1 mM H_2_O_2_ and 400 µL of enzyme extract. The absorbance of the mixture was read at 290 nm against the blank and the enzyme activity was expressed in units mg^−1^ protein.

#### Total seed phenolics content

Total seed phenolics were determined using Folin-Ciocalteu method^32^ with some modifications. The extracts were mixed with 5 mL Folin-Ciocalteu reagent (previously diluted with water 1:10 v/v) and 4 mL (75 g/L) of sodium carbonate. For 15 sec, the tubes were vortexed and allowed to stand for 30 min at 40 °C for colour development. Then absorbance of the triturent was read at 755 nm using the spectrophotometer. Total phenolic content were expressed as mg/kg of tannic acid equivalent based on the calibration curve:

#### Total flavonoid content

Total flavonoid content was determined spectrophotometrically by using the method of^33^ The standard curve for the total flavonoids was made using different concentrations of rutin (0–100 mg/L) as standard solution. Then the total flavonoids wre expressed as milligrams of rutin equivalents per g of dried fraction.

#### Yield attributes

Different yield attributes such as total No. of tiller/plant, spike length, No. of spikelet/spike, no. of seeds per plant, 1000 grain weight, days to headings and maturity were calculated.

### Tocopherol analysis

#### Seed lipid extraction

For the determination of tocopherol in grains of wheat, the oil was extracted from the grains as described by^34^. Following the method, 20 g of grains (powdered form) were placed in cotton thumb-bell, then added 250 mL *n*-hexane in a round bottom flask. Then the extraction of oil was done at 70 °C in Soxhlet apparatus. The crud oil was separated from the solvent using a rotary evaporator (Buchi, Rotovapor R-421, Switzerland) and used for the analysis of tocopherols.

#### Seed oil tocopherol determination (α, γ, δ)

The HPLC system was equipped with S-1122 dual piston solvent delivery system, and S-3210 UV/VIS diode array detector. Twenty μL of the extract was injected into the Hypersil ODS reverse phase (C-18) column (5 μm particle size, 250 mm × 4.6 ID Themohypersil GmbH, (Darmstadt, Germany) fitted with a C18 guard column and methanol:acetonitrile: methylene chloride (50:44:6, *v*/*v*) mobile phase at 1 mL/min flow rate. The peak areas were recorded and calculated by a computer with SRI peak simple chromatography data acquisition and integration software (SRI instrument, Torrance, CA, USA) at 295 nm. The quantification of tocopherols was done by comparing the samples with pure standards purchased from Sigma-Aldrich (Buchs, Switzerland).

### Statistical analysis

Analysis of variance (ANOVA) was carried out using computer software “CoStat window version 6.2” (CoHort Software, 2003, Monterey, CA, USA). Students Newman keul test was applied to test the significance of difference among mean values. The correlation studies and PCA analysis were done using XLSTAT software.

## Results

**Figure 1:**
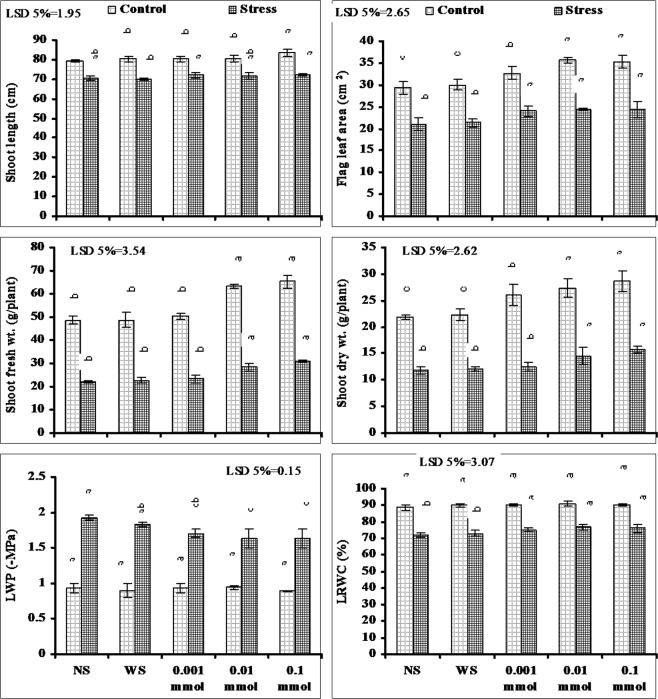
Different growth attributes and leaf water relations of drought-stressed wheat plants sprayed with different levels of α-tocopherol when applied at the heading stage (mean ± SE).

The present study was conducted to find out the responses of drought stressed wheat plants to varying doses of α-Toc in terms of growth, physio-biochemical, seed yield and nutritional quality. Data presented in Fig. 1 for varying growth attributes shows that drought stress significantly reduced the shoot length, flag leaf area (FLA), and fresh and dry masses of wheat plants. Exogenous application of α-Toc did not show any increasing or decreasing effect on shoot length under water stress, but under non-stressed conditions a small increase was found only in plants supplied with 0.1 mmol level of α-Toc. However, the FLA increased significantly both under stressed and non-stressed conditions due to foliar spray of α-Toc and all the levels were found equally effective in this regard under water stressed conditions. Likewise the shoot fresh, the dry masses also increased significantly due to foliar spray of α-Toc both under water stressed and non-stressed conditions and the higher levels of α-Toc (0.01 and 0.1 mmol) were found more effective as compared with 0.001 mmol level. The maximum increase in shoot fresh and dry weights under water deficit conditions was 29.03% and 24.50% respectively when plants were supplied with 0.1 mmol level of α-Toc (Fig. 1).

Adverse effects of water stress were also found on the leaf water potential (Ψ_w_) and leaf relative water content (LRWC). A significant decrease was found in these attributes due to imposition of water stress. Exogenous application of different regimes of α-Toc found effective in reducing the adverse effects of drought on these attributes. The maximum amelioration in Ψ_w_ was found in plants that were sprayed with 0.01 and 0.1 mmol levels of α-Toc as compared with 0.001 mmol level. However, in relation with LRWC all applied α-Toc levels were found equally effective in reducing the adverse effects of water stress (Fig. 1).

Plant fertile tillers, spike length and number of spikelets decreased significantly due to imposition of water stress. Foliar spray of different levels of α-Toc significantly increased the plant fertile tillers, spike length and number of spikelets/spike both under non-stressed and water stressed conditions. A gradual increase was recorded in plant fertile tillers, spike length and number of spikelets/spike with an increase in α-Toc levels (Table 1).

**Table 1:** Different yield attributes of drought-stressed wheat plants sprayed with different levels of α-tocopherol when applied at the heading stage (mean ± SE).

α-Toc (m mol)	Fertile tillers per plant		Spike length (cm)		Spikelet/spike		Number of grains/plant		100 grain wt (g)		yield/plant (g)		Days to Heading		days to maturity	
	Control	Stress	Control	Stress	Control	Stress	Control	Stress	Control	Stress	Control	Stress	Control	Stress	Control	Stress
NS	6.33 ± 0.33^c^	3.33 ± 0.33^c^	9.35 ± 0.05^b^	6.65 ± 0.32^c^	16 ± 0.6^c^	12 ± 0.6^d^	700 ± 17^c^	300 ± 16^c^	4.23 ± 0.23^d^	2.92 ± 0.04^b^	28.00 ± 0.69^c^	12.00 ± 0.23^c^	104 ± 1^c^	80 ± 1^c^	131 ± 1^b^	103 ± 3^c^
WS	7.33 ± 0.66^b^	3.67 ± 0.33^b^	9.32 ± 0.02^b^	6.70 ± 0.35^c^	16 ± 0.3^c^	12 ± 0.3^d^	743 ± 17^b^	313 ± 17^bc^	4.67 ± 0.03^c^	2.98 ± 0.02^b^	29.73 ± 3.27^b^	12.53 ± 0.27^cb^	108 ± 4^c^	83 ± 2^bc^	131 ± 2^b^	113 ± 2^b^
0.001	8.00 ± 0.57^b^	4.33 ± 0.33^b^	9.50 ± 0.01^b^	7.30 ± 0.06^b^	17 ± 0.6^b^	14 ± 0.3^c^	766 ± 23^ab^	346 ± 13^ab^	4.93 ± 0.14^bc^	3.10 ± 0.15^b^	30.67 ± 1.33^ab^	13.87 ± 0.63^ab^	113 ± 2^b^	90 ± 2^b^	130 ± 1^b^	114 ± 3^a^
0.01	9.00 ± 0.66^a^	4.67 ± 0.33^a^	9.93 ± 0.12^b^	7.60 ± 0.06^ab^	18 ± 0.3^a^	14 ± 0.3^b^	780 ± 20^ab^	346 ± 13^ab^	4.95 ± 0.16^bc^	3.33 ± 0.33^ab^	31.20 ± 2.28^ab^	13.87 ± 0.73^ab^	116 ± 3^ab^	100 ± 2^a^	136 ± 3^a^	114 ± 2^a^
0.1	9.67 ± 0.66^a^	5.33 ± 0.33^a^	9.93 ± 0.07^b^	7.67 ± 0.05^a^	18 ± 0.3^a^	15 ± 0.3^a^	800 ± 18^a^	356 ± 19^a^	5.60 ± 0.10^a^	3.50 ± 0.29^a^	32.00 ± 1.15^a^	14.27 ± 1.18^a^	118 ± 2^a^	100 ± 2^a^	138 ± 2^a^	118 ± 2^a^
**LSD 5%**	**0.91**		**0.35**		**0.76**		**39.94**		**0.38**		**1.60**		**4.26**		**4.56**	

Values with same alphabets in superscript in a column do not differ significantly.

A significant decrease in number of grains/plant, 100 grain weight and grain yield/plant due to imposition of water stress. Exogenous application of different levels of α-Toc as foliar spray significantly reduced the adverse effects of water stress on these yield attributes. The increase in these yield attributes increased with an increase in α-Toc level and the maximum increase was recorded at maximum level of α-Toc (Table 1).

Days to heading and days to plant maturity of wheat plants were adversely effected due to imposition of water stress. A significant ameliorating effect of exogenously applied α-Toc was found on these attributes. The maximum amelioration was found in plants that were supplied with 0.1 m mol level of α-Toc (Table 1).

**Figure 2:**
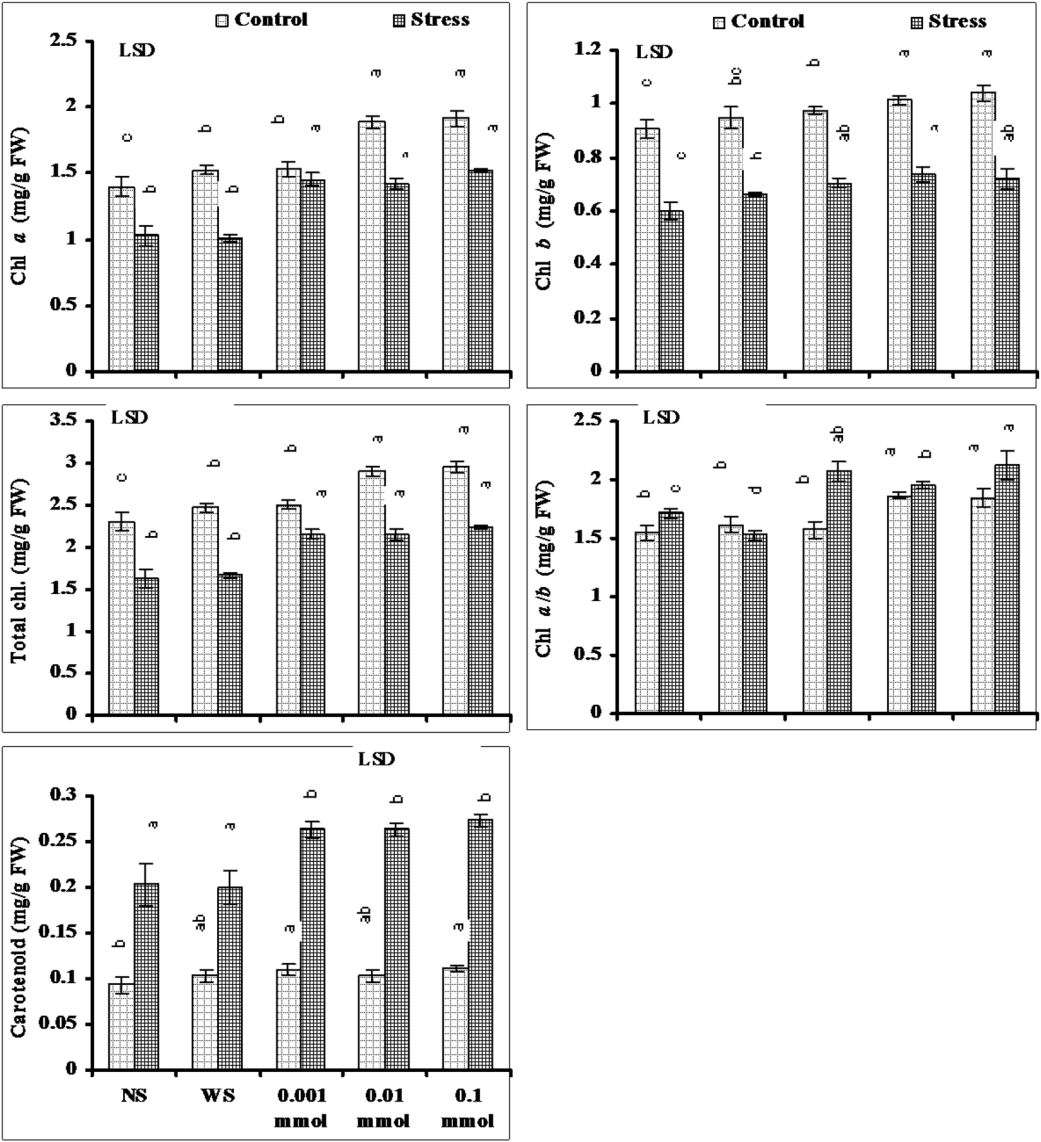
Leaf photosynthetic pigments of drought-stressed wheat plants sprayed with different levels of α-tocopherol when applied at the heading stage (mean ± SE).

Data presented in Fig. 2 shows that leaf Chl. *a*, *b*, and total Chl. contents decreased significantly due to water stress. Foliar spray of different regimes of α-Toc significantly increased the Chl. *a*, *b* and total Chl. contents both under stressed and non-stressed conditions. However, the effective levels were different under water stressed and non-stressed conditions. Under water deficit conditions all three levels of α-Toc were found equally effective in increasing the Chl. *a*, *b* and total Chl. contents. However, under non-stressed conditions this increase in Chl. *a*, *b* and total Chl. was maximum in plants sprayed with 0.01 and 0.1 mmol levels of α-Toc (Fig. 2).

Drought stress significantly increased the Chl. *a/b* ratio and leaf carotenoid content of wheat plants. Foliar spray of different regimes of α-Toc further enhanced the Chl. *a*/*b* and leaf carotenoid content both under water stress and non-stressed conditions. Under non-stressed conditions this increase in Chl *a*/*b* ratio was found in plants sprayed with 0.01 and 0.1 mmol levels of α-Toc. However, under water stressed conditions 0.001 and 0.1 mmol levels were found more effective in increasing the Chl *a*/*b* ratio. The increase in leaf carotenoid due to α-Toc foliar application was similar at all levels both under stressed and non-stressed conditions (Fig. 2).

**Figure 3:**
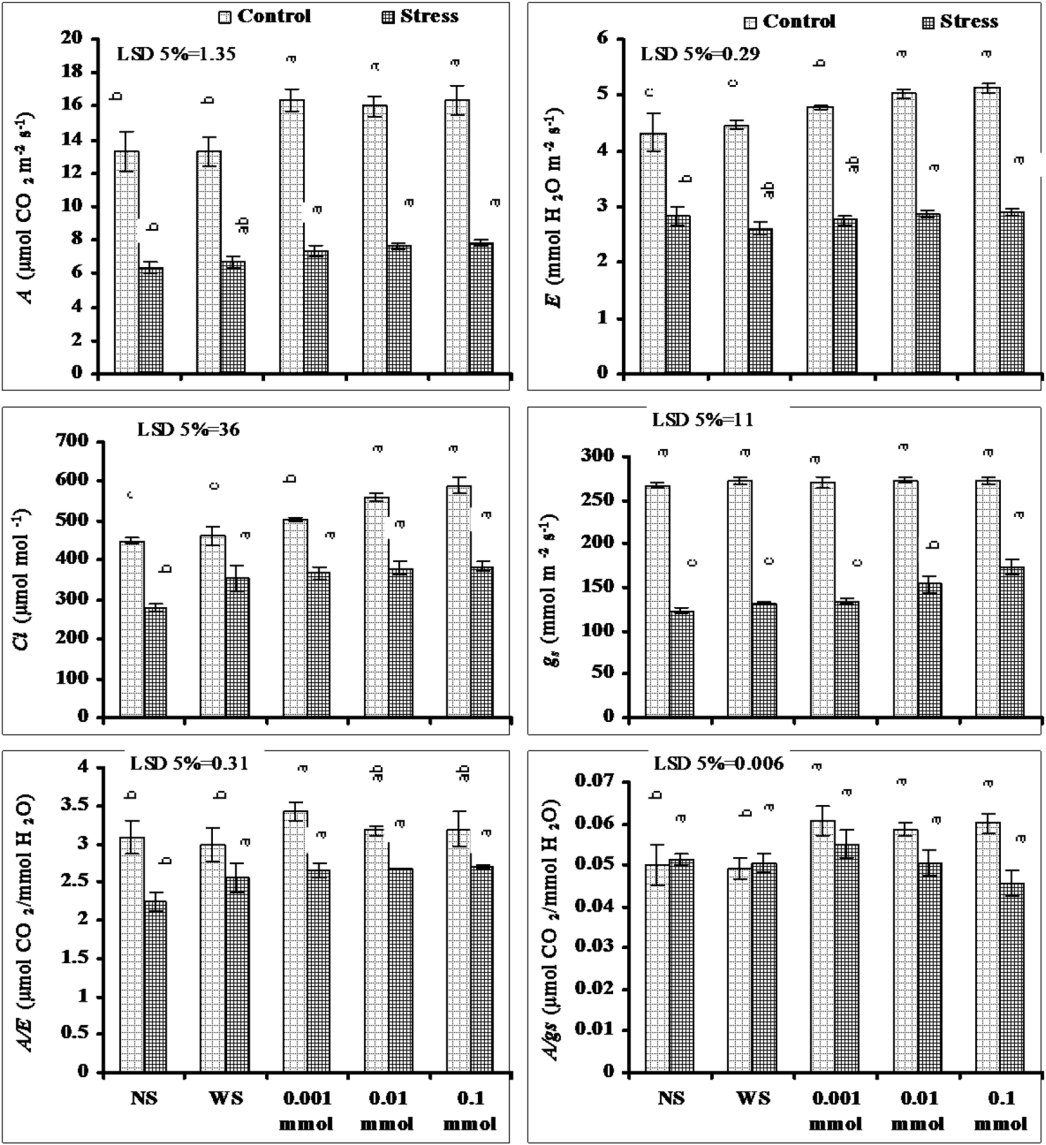
Leaf gas exchange attributes of drought-stressed wheat plants sprayed with different levels of α-tocopherol when applied at the heading stage (mean ± SE).

Significant reduction was recorded in different gas exchange attributes such as net photosynthetic rate (*A*), transpiration rate (*E*), intrinsic CO_2_ (*Ci*) and stomatal conductance (*gs*) due to water stress application. Foliar spray of different levels of α-Toc found effective in reducing the adverse effects of water stress on these gas exchanges attributes. All applied α-Toc levels were found equally effective in decreasing the adverse effects of drought on these attributes except to that of *g*_*s*_ where the maximum amelioration was found in plants sprayed with 0.1 m mol level of α-Toc (Fig. 3).

Water use efficiency (*A*/*E*) of wheat plants also decreased significantly but the intrinsic water use efficiency (*A*/*gs*) remained unaffected due to water stress. Foliar spray of different levels of α-Toc found effective in improving the *A*/*E* and *A*/*g*_*s*_ both under stressed and non-stressed conditions. In case of *A*/*E* under water stress all the levels of α-Toc found equally effective but under non-stressed conditions 0.001 m mol level was the most effective one. However, the improvement in *A*/*gs* under water stress was only in plants sprayed with 0.001 m mol level of α-Toc but under non-stressed conditions all the applied levels of α-Toc increased the *A*/*gs* being maximum at 0.001 m mol level (Fig. 3).

**Figure 4:**
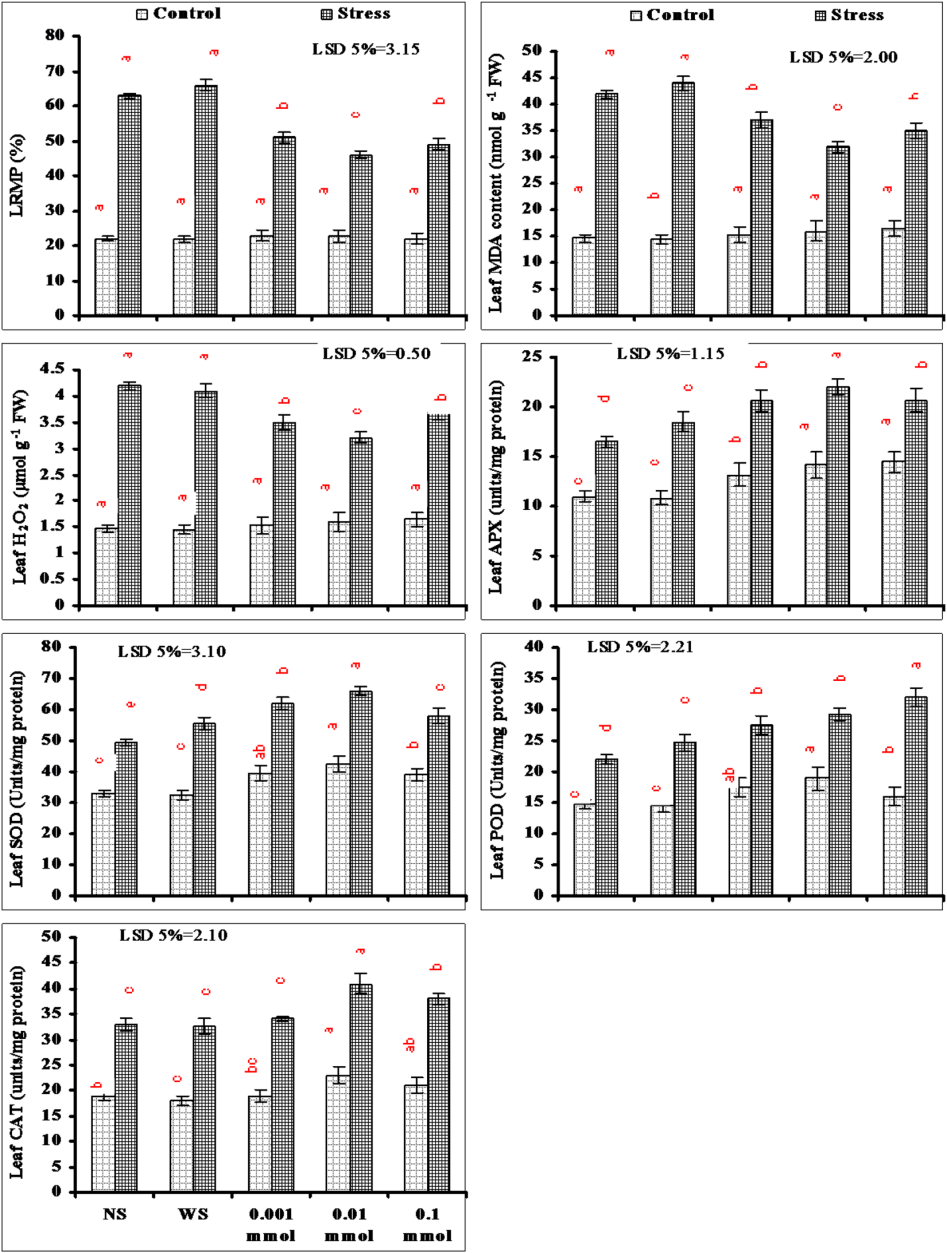
Lipid peroxidation and enzymatic antioxidative defence mechanism of drought-stressed wheat plants sprayed with different levels of α-tocopherol when applied at the heading stage (mean ± SE).

Data presented in Fig. 4 shows that drought stress significantly enhanced the LRMP, MDA and H_2_O_2_ content showing an increase in oxidative stress and its damages on membranes. Foliar application of different levels of α-Toc found effective in reducing the adverse effects of oxidative damages. A decrease in LRMP, MDA and H_2_O_2_ contents was recorded and the maximum reduction was found in plants that were sprayed with 0.01 mmol level of α-Toc as compared with other levels (Fig. 4).

Leaf SOD, POD, APX and CAT activities of wheat plants increased significantly under water deficit conditions. Exogenous application of different levels of α-Toc as foliar spray further enhanced the activities of the studied antioxidant enzymes. In case of SOD, APX and CAT the maximum increase in the activity was found in plants sprayed with 0.01 mmol level of α-Toc under water deficit conditions. However, in case of POD this increase was found in plants sprayed with 0.1 mmol level of α-Toc both under stressed and non-stressed conditions (Fig. 4).

**Figure 5:**
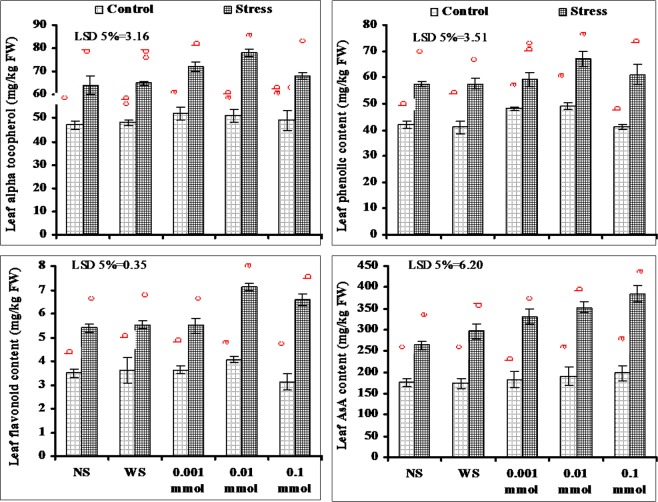
Contents of leaf non-enzymatic compounds of drought-stressed wheat plants sprayed with different levels of α-tocopherol when applied at the heading stage (mean ± SE).

Among different studied leaf non-enzymatic antioxidants, the content of α-Toc, phenolics, flavonoids and AsA increased significantly in plants grown under water deficit conditions. Foliar spray of different levels of α-Toc further enhanced the contents of all these biochemicals but the extent of increase was foliar dose specific. In case of α-Toc, phenolics and flavonoids the maximum increase due to α-Toc foliar spray was found in plants supplied with 0.01 mmol level. However, in case of leaf AsA content, α-Toc-applied this increase was maximum in plants supplied with 0.1 mmol as compared with other levels (Fig. 5).

**Figure 6:**
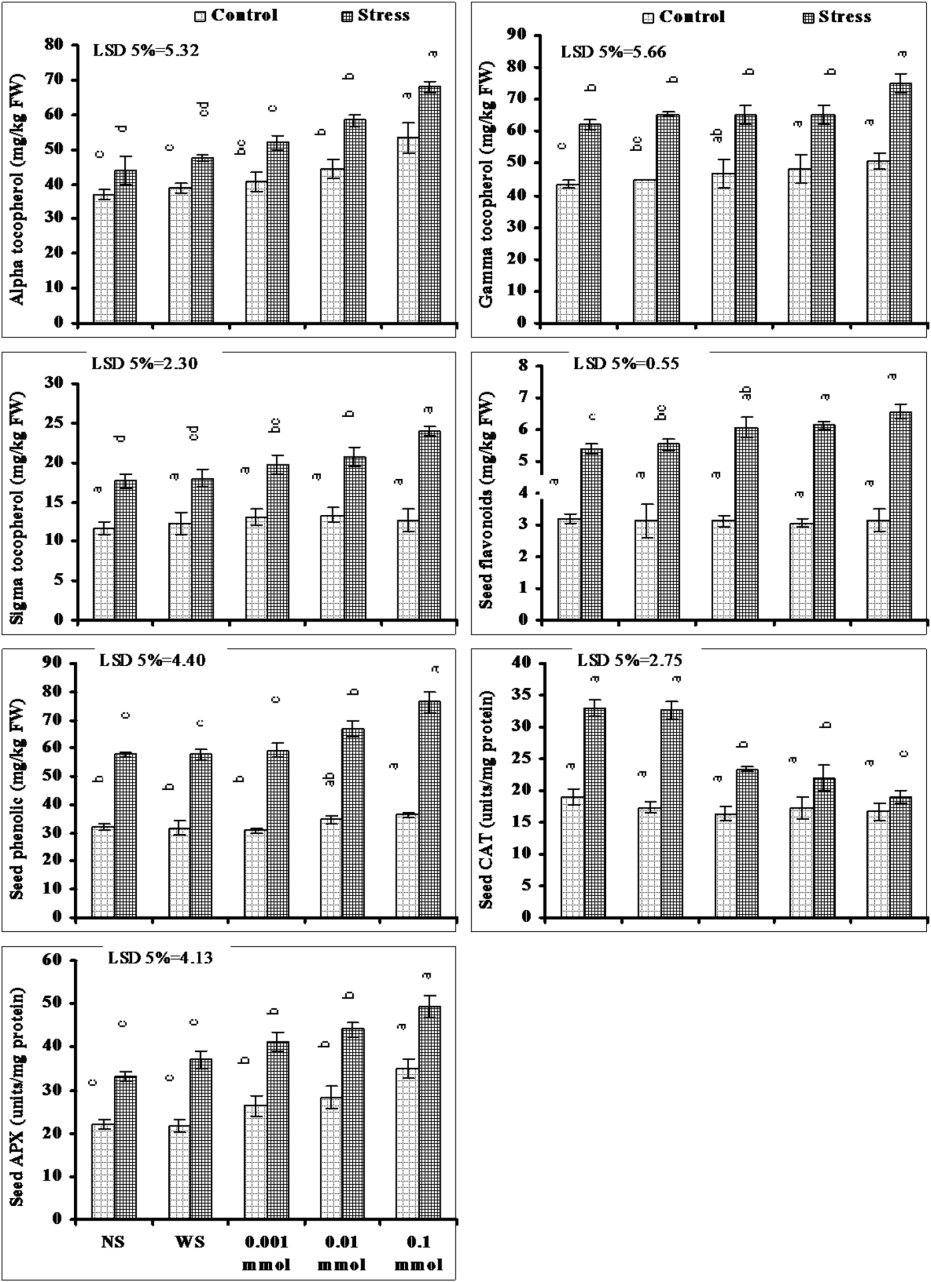
Seed nutritional quality of drought-stressed wheat plants sprayed with different levels of α-tocopherol when applied at the heading stage (mean ± SE).

Seed flavonoids and phenolic contents also increased due to water stress. Exogenous application of different levels of α-Toc further enhanced the seed flavonoids and phenolic contents both under stressed and non-stressed conditions except to that of seed flavonoids that remained unaffected due to foliar spray of different levels of α-Toc under non-stressed conditions (Fig. 6).

Drought stress significantly increased the seed CAT and APX activities. A marked decrease in seed CAT activity was recorded due to foliar spray of α-Toc on wheat plants under water stressed conditions and the maximum decrease was found at the maximum level of α-Toc. However, the seed APX activity was further increased due to foliar spray of α-Toc under both stressed and non-stressed conditions (Fig. 6).

Seed α-Toc content increased significantly due to water stress. Exogenous application of different levels of α-Toc as foliar spray further increased the seed α-Toc content both under water stressed and non-stressed conditions (Fig. 6). The maximum increase in α-Toc due to its foliar application was found at its maximum level (0.1 mmol).

A significant increase in seed γ- and δ-toc was found under drought stress. Exogenous application of different levels of α-Toc significantly increased the seed γ- and δ-toc. Alpha tocopherol applied this increase in seed γ-toc was only found at its maximum level under water stressed conditions but under non-stressed conditions seed γ-toc content increased gradually with the increase in α-Toc levels. However, the seed δ-Toc content increased with an increase in α-Toc levels under water deficit conditions but under non-stressed conditions seed δ-Toc contents remain unaffected due to foliar-applied α -Toc at all applied levels (Fig. 6).

### Correlations and PCA analysis

**Figure 7:**
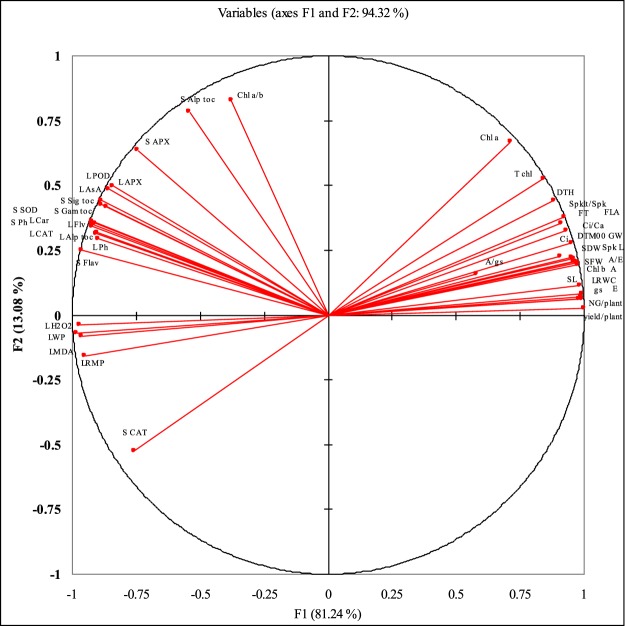
Principle component analysis of the studied attributes of the drought-stressed wheat plants sprayed with different levels of α-tocopherol when applied at the heading stage (mean ± SE).

PCA analysis presented in Fig. 7 shows the correlation studies of various studied attributes. Of the extracted components F1 (81.24%) and F2 (13.08%) has major contribution with a total contribution of 94.32% to find out the significant correlations among different studied attributes. Factor F1 has divided the studied attributes in three distinct classes as encircled in the Fig. The major class encircled shows a strong relation among different gas exchange (*A*, *E*, *C*^*i*^, *g*_*s*_, *A*/*E*, *Ci*/*Ca*, and *A*/*g*_*s*_), photosynthetic pigments (Chl. *a*, Chl. *b* and T Chl.) growth (SFW, SDW, SL, FLA) and yield attributes (DTM, 100 GW, NG/plant, yield/plant, FT, Spkt/Spk) was found and the 2^nd^ class shows a strong relation among different enzymatic (L SOD, L POD, L CAT, L APX, S CAT, S SOD) and non-enzymatic (L ph, L Flv, L AsA, L Car, L Alp Toc, S Alp Toc, S Gam Toc, S Sig toc, S Flv, S Ph,) antioxidants in leaf and seed. While in the third class the parameters regarding lipid peroxidation (LWP, LRMP, LMDA and LH_2_O_2_) are grouped together and has clear negative relation with the most of the studied attributes. The complete correlations among all studied attributes (Pearson coefficient correlations) along with the level of significance are presented in Table 2.

**Table 2:** Pearson correlation coefficients (*r*^4^) among different studied attributes of water stressed wheat plants exogenously supplied with different levels of alpha tocopherols at heading stage.

Variables	SFW	SDW	LRWC	LWP	Chl a	Chl b	Car.	*A*	*E*	*Ci*	*gs*	*A/E*	*A/gs*	L Alp toc
SFW	**1**	0.988***	0.950***	−0.939***	0.836***	0.976***	−0.834***	0.968***	0.980***	0.967***	0.948***	0.877***	0.589***	−0.832***
SDW	0.988***	**1**	0.959***	−0.948***	0.820***	0.984***	−0.832***	0.990***	0.987***	0.966***	0.957***	0.923***	0.636***	−0.825***
LRWC	0.950***	0.959***	**1**	−0.997***	0.739***	0.981***	−0.883***	0.975***	0.974***	0.908***	0.993***	0.928***	0.516***	−0.869***
LWP	−0.939***	−0.948***	−0.997***	**1**	−0.737***	−0.977***	0.868***	−0.963***	−0.960***	−0.902***	−0.992***	−0.930***	−0.480***	0.864***
Chl a	0.836***	0.820***	0.739***	−0.737***	**1**	0.828***	−0.416*	0.766***	0.760***	0.888***	0.724***	0.746***	0.543***	−0.446*
Chl b	0.976***	0.984***	0.981***	−0.977***	0.828***	**1**	−0.828***	0.984***	0.977***	0.970***	0.969***	0.943***	0.601***	−0.813***
Chl a/b	−0.191 ns	−0.220 ns	−0.331 ns	0.320 ns	0.356 ns	−0.223 ns	0.669***	−0.304 ns	−0.311 ns	−0.091 ns	−0.335 ns	−0.244 ns	−0.098 ns	0.580***
T chl	0.922***	0.915***	0.859***	−0.856***	0.978***	0.926***	−0.585***	0.878***	0.872***	0.955***	0.845***	0.849***	0.587***	−0.600***
Car	−0.834***	−0.832***	−0.883***	0.868***	−0.416	−0.828***	**1**	−0.871***	−0.897***	−0.730***	−0.880***	−0.744***	−0.484***	0.965***
*A*	0.968***	0.990***	0.975***	−0.963***	0.766***	0.984***	−0.871***	**1**	0.991***	0.944***	0.965***	0.945***	0.663***	−0.851***
*E*	0.980***	0.987***	0.974***	−0.960***	0.760***	0.977***	−0.897***	0.991***	**1**	0.932***	0.966***	0.896***	0.631***	−0.883***
*Ci*	0.967***	0.966***	0.908***	−0.902***	0.888***	0.970***	−0.730***	0.944***	0.932***	**1**	0.895***	0.911***	0.658***	−0.720***
*gs*	0.948***	0.957***	0.993***	−0.992***	0.724***	0.969***	−0.880***	0.965***	0.966***	0.895***	**1**	0.919***	0.449*	−0.888***
*A/E*	0.877***	0.923***	0.928***	−0.930***	0.746***	0.943***	−0.744***	0.945***	0.896***	0.911***	0.919***	**1**	0.612***	−0.723***
Ci/Ca	0.967***	0.966***	0.908***	−0.902***	0.888***	0.970***	−0.730***	0.944***	0.932***	1.000***	0.895***	0.911***	0.658***	−0.720***
A/gs	0.589***	0.636***	0.516**	−0.480	0.543**	0.601***	−0.484**	0.663***	0.631***	0.658***	0.449*	0.612***	**1**	−0.387*
LMDA	−0.915***	−0.926***	−0.987***	0.990***	−0.731***	−0.956***	0.837***	−0.943***	−0.941***	−0.864***	−0.981***	−0.910***	−0.445*	0.823***
L RMP	−0.919***	−0.928***	−0.981***	0.986***	−0.781***	−0.960***	0.797***	−0.939***	−0.935***	−0.881***	−0.973***	−0.915***	−0.458*	0.792***
L H2O2	−0.910***	−0.921***	−0.990***	0.992***	−0.710***	−0.960***	0.860***	−0.946***	−0.942***	−0.869***	−0.975***	−0.917***	−0.483**	0.833***
L AsA	−0.772***	−0.776***	−0.844***	0.825***	−0.355 ns	−0.776***	0.984***	−0.830***	−0.861***	−0.657***	−0.828***	−0.686***	−0.511**	0.937***
L Flav	−0.805***	−0.809***	−0.848***	0.844***	−0.458*	−0.811***	0.943***	−0.854***	−0.876***	−0.729***	−0.838***	−0.734***	−0.567***	0.954***
L Ph	−0.810***	−0.798***	−0.860***	0.867***	−0.466*	−0.812***	0.930***	−0.826***	−0.861***	−0.718***	−0.867***	−0.706***	−0.384*	0.972***
L Alp toc	−0.832**	−0.825***	−0.869***	0.864***	−0.446*	−0.813***	0.965***	−0.851***	−0.883***	−0.720***	−0.888***	−0.723***	−0.387*	**1**
S SOD	−0.758***	−0.757***	−0.834***	0.832***	−0.345 ns	−0.751***	0.951***	−0.793***	−0.832***	−0.619***	−0.854***	−0.657***	−0.295 ns	0.982***
L POD	−0.735***	−0.730***	−0.809***	0.799***	−0.308	−0.737***	0.966***	−0.780***	−0.822***	−0.614***	−0.796***	−0.626***	−0.440*	0.933***
L CAT	−0.802***	−0.816***	−0.890***	0.887***	−0.435*	−0.830***	0.964***	−0.869***	−0.883***	−0.718***	−0.885***	−0.776***	−0.482**	0.967***
L APX	−0.709***	−0.710***	−0.813***	0.806***	−0.274 ns	−0.714***	0.955***	−0.762***	−0.799***	−0.565**	−0.823***	−0.630***	−0.289 ns	0.960***
S CAT	−0.779***	−0.803***	−0.822***	0.840***	−0.869***	−0.842***	0.468**	−0.786***	−0.753***	−0.810***	−0.825***	−0.855***	−0.335 ns	0.512**
S APX	−0.569**	−0.574**	−0.716***	0.700***	−0.118 ns	−0.597***	0.906***	−0.654***	−0.687***	−0.427*	−0.700***	−0.533**	−0.298 ns	0.848***
S Alp toc	−0.336 ns	−0.346 ns	−0.500**	0.477**	0.118 ns	−0.374*	0.770***	−0.451*	−0.479**	−0.205 ns	−0.466*	−0.338 ns	−0.291 ns	0.678***
S gam toc	−0.786***	−0.791***	−0.894***	0.883***	−0.423	−0.821***	0.953***	−0.851***	−0.870***	−0.689***	−0.867***	−0.754***	−0.489**	0.886***
S gam toc	−0.771***	−0.768***	−0.842***	0.829***	−0.366	−0.784***	0.973***	−0.824***	−0.852***	−0.676***	−0.816***	−0.695***	−0.536**	0.910***
S flav	−0.885***	−0.889***	−0.934***	0.919***	−0.528**	−0.892***	0.988***	−0.926***	−0.946***	−0.800***	−0.921***	−0.809***	−0.559**	0.948***
S Ph	−0.802***	−0.813***	−0.890***	0.876***	−0.436*	−0.835***	0.970***	−0.874***	−0.888***	−0.725***	−0.863***	−0.775***	−0.575**	0.914***
DTH	0.934***	0.948***	0.916***	−0.915***	0.906***	0.956***	−0.655***	0.921***	0.902***	0.952***	0.914***	0.920***	0.523**	−0.651***
FT	0.981***	0.986***	0.929***	−0.919***	0.889***	0.977***	−0.755***	0.962***	0.960***	0.984***	0.923***	0.900***	0.635***	−0.762***
Spk L	0.975***	0.979***	0.988***	−0.985***	0.825***	0.991***	−0.828***	0.977***	0.976***	0.946***	0.983***	0.932***	0.543**	−0.825***
Spkt/Spk	0.967***	0.970***	0.940***	−0.934***	0.912***	0.976***	−0.718***	0.950***	0.940***	0.970***	0.934***	0.921***	0.573**	−0.717***
NOG/plant	0.962***	0.972***	0.993***	−0.986***	0.730***	0.981***	−0.910***	0.987***	0.990***	0.917***	0.985***	0.917***	0.575**	−0.899***
100 G Wt	0.977***	0.989***	0.948***	−0.941***	0.824***	0.981***	−0.812***	0.975***	0.976***	0.964***	0.945***	0.904***	0.624***	−0.815***
yield/plant	0.962***	0.972***	0.993***	−0.986***	0.730***	0.981***	−0.910***	0.987***	0.990***	0.917***	0.985***	0.917***	0.575**	−0.899***
DTM	0.964***	0.955***	0.959***	−0.964***	0.822***	0.980***	−0.805***	0.946***	0.941***	0.963***	0.960***	0.922***	0.496**	−0.820***

## Discussion

In order to overcome the adverse effects of drought different strategies are being applied including exogenous application of different chemicals through different modes. The use of these compounds as foliar spray is one of the promising one because exogenously/foliary applied these compounds are readily absorbed by leaf and translocated to different plant parts where they play different roles in cellular metabolism^4^. Exogenously applied these compounds after translocation in specific plant parts also regulate its own metabolism^4,5^. Among exogenously applied compounds the effective ones are those which give better yield and are of interest for agricultural scientists. Reports depict that the effective outcomes through the exogenous application of organic compounds is plant species, cultivar within the species and growth stage specific. Among different chemicals the exogenous use of lipophilic antioxidative compounds is increasing due to their effective roles. Among these the interest in α–toc use is increasing. Studies reveal that exogenous application of α-tochopherol as foliar spray found effective for the induction of stress tolerance in different crop plants^35^. These studies depict its role in yield and physiological attributes but little is reported about its role in yield increments in relation with antioxidative defense mechanism and photosynthetic attributes. These attributes severely effects under water deficit conditions. In present study, drought stress significantly reduced the biomass production of wheat plants that was associated with the decrease in photosynthetic activity, water relations, photosynthetic pigments and increased lipid peroxidation.

In present study water stress significantly reduced the plant biomass production, seed yield and seed nutritional quality of wheat plant. This reduction in biomass production and yield attributes were associated with the reduction in leaf photosynthetic pigments, gas exchange, attributes, water relations and oxidative defense mechanism but the negative was true for lipid peroxidation. Such negative effects of water stress on studied attributes is a well understood mechanism as depicted in plethora of literature^4,36^. However, in present study exogenous application of different doses of α-Toc significantly improved the wheat growth, yield and seed nutritional quality. This improvement in these attributes due to α-Toc exogenous application might be due to its involvement/role in different physio-biochemical attributes possibly due to its translocation to different plant parts after its application, where it played a significant role in plant stress tolerance^8^. Furthermore, in present study exogenous application of α-Toc improved the seed α-Toc content and the contents of other members of seeds α-Toc. This increase in seed α-Toc content increased with increasing its exogenous concentration that shows its long term translocation to different plant parts and this increase in the content of α-Toc also shows its involvement in the regulation of its own metabolism as reported earlier by^8^ and by other researchers for different exogenously applied compounds^4–6^.

It is well known that plant biomass production and seed yield are directly linked to the plant photosynthetic activity and assimilation, which is linked to plant water relations as well as plant photosynthetic pigments. These physiological attributes directly affect the plant photosynthetic efficiency by affecting light capturing capacity and assimilation. In present study, improvement in wheat growth and yield due to α-Toc exogenous fertigation is associated with the improvement in plant water relations leaf photosynthetic pigments (chl, *a*, *b*, total chlorophyll, and carotenoids), and improved plant photosynthetic activity in terms of *A, E, Ci, g*_*s*_*, A/E* and *A/gs*. The first and the foremost is the functioning of proper water relations that directly influences the plant photosynthesis as focused in present study in plants fertigated with α-Toc. This might be due to the involvement of α-Toc in cellular osmotic adjustment by playing its significant role in H-ATPase system being an integral part of cellular membranes^3^. It has been reported that α-Toc application significantly improved the water stress tolerance of sunflower plants by improving the plant water relations and played a significant role in cellular osmotic adjustment that was associated with the more absorption of nutrients from soil solution^16^. Furthermore, this improvement in plant water relations due to exogenous application of α-Toc is also associated with the improved nitrogen metabolism that alternately increased the proline accumulation, plant growth and yield components^37^. These findings can be correlated with the results of present study where α-Toc foliar application on water stressed wheat plants clearly improved the different components of plant water relations along with plant photosynthetic attributes that are directly linked to each other as a result better growth and yield. Secondly, being an integral part of cellular membrane, it play a role in reducing the degradation of photosynthetic pigment under stress-full condition as a better maintenance of light capturing ability^14^ and better photosynthetic activity as found in present study. Furthermore, it has also been found that α-Toc also protects the D1 protein of photosynthetic units from the oxidative damage under stressful environment^38^ and also the chloroplast membranes.

### Roles of tocopherol in water stress

In present study, exogenous application of α-Toc showed long term translocation that might have played a significant role in the improvement of plant photosynthetic activity as a result better assimilation, growth and final production. It was found earlier by^35^ and^39^ that α-Toc foliar application enhanced the assimilation process in terms of better carbohydrates and protein biosynthesis^40^. These findings can be correlated with the present findings, where α-Toc foliar application also enhanced the plant biomass production and seed yield.

The better production is also linked with the better antioxidative defensive mechanism that is of prime importance under stressful environmental conditions for the better functioning of cellular membranes. In present study α-Toc foliar application maintained a better antioxidative defense mechanism in terms of the increased activities of enzymatic antioxidant and the content of non-enzymatic compounds with the lowered MDA content. Furthermore, the wheat plants supplied with α-Toc maintained a better level of leaf carotenoid content that have a confirmed role in photosynthesis as accessory pigments as well as an antioxidative components, that is associated with the better growth and production as found in present study. These findings further confirm the role of α-Toc in better photosynthesis and improved antioxidative defense mechanism as a result better growth and production. Furthermore α-Toc itself acts as an ROS quencher. It is an integral part of membranes as it is a best antioxidant. The reduced lipid peroxidation in α-Toc supplied wheat plants might be due to its enhanced contents in cell after its application and translocation.

In earlier studies it was found that exogenous application of different antioxidative compounds significantly improved the seed nutritional quality in terms antioxidant enzyme and non-enzymatic antioxidative compounds^4,41^. In present study α-Toc foliar application also increased the nutritional quality in terms of its own content in wheat seeds. It was found exogenous application of biochemicals/antioxidants not only regulates its own metabolism but also enhances the content of other antioxidative enzymes^5,42^ and the content of non-enzymatic compounds^41^. Similar has been found in present study where α-Toc foliar application not only improved its metabolism in terms of increased content of α-Toc and γ-Toc but also other studied enzymatic and non-enzymatic compounds, as a result better seed nutritional quality in terms of antioxidative properties.

## Conclusion

In conclusion it was found that α-Toc foliar application improved the wheat drought tolerance in terms of better biomass production, seed yield and nutritional quality that is associated with its better role in improvement of plant water relations, photosynthetic activity and antioxidative defense mechanism. Among different applied levels of α-Toc the higher levels (0.01 and 0.1 m Mol) were found better for the drought tolerance of wheat plants in terms of better growth and seed yield and nutritional quality. On average basis 15.09% increase in grain yield was recorded. On hectare basis an increase of 556.5 kg was recoded due to α-Toc foliar application under water deficit conditions that counts an economic benefit of 17298 Rs. according to local market prices^43–46^.
